# A highly conserved ABC transporter mediates cello-oligosaccharide uptake in the extremely thermophilic, lignocellulolytic bacterium *Anaerocellum bescii* (f. *Caldicellulosiruptor bescii*)

**DOI:** 10.1128/aem.01284-25

**Published:** 2025-12-18

**Authors:** Hansen Tjo, Virginia Jiang, Anherutowa Calvo, Jerelle A. Joseph, Jonathan M. Conway

**Affiliations:** 1 Department of Chemical and Biological Engineering, Princeton University 6740, Princeton, New Jersey, USA; 2 Omenn-Darling Bioengineering Institute, Princeton University 6740, Princeton, New Jersey, USA; 3 Andlinger Center for Energy and the Environment, Princeton University 6740, Princeton, New Jersey, USA; 4 Department of Chemistry, Princeton University, Princeton, New Jersey, USA; 5 Princeton Institute for Computational Science and Engineering, Princeton University, Princeton, New Jersey, USA; 6 Molecular Biology Department, Princeton University 6740, Princeton, New Jersey, USA; 7 High Meadows Environmental Institute, Princeton University 6740, Princeton, New Jersey, USA; University of Nebraska-Lincoln, Lincoln, Nebraska, USA

**Keywords:** thermophile, biotechnology, cellulose, cello-oligosaccharide, ABC sugar transporter, substrate-binding protein, *Caldicellulosiruptor*, *Anaerocellum bescii*, genetics

## Abstract

Cellulose deconstruction and utilization are foundational to renewable biofuel and biochemical production. *Anaerocellum bescii* (formerly *Caldicellulosiruptor bescii*) is an extremely thermophilic cellulolytic bacterium, notable for its multi-domain cellulases and hemicellulases that efficiently degrade lignocellulosic biomass. However, the mechanisms by which *A. bescii* transports cello-oligosaccharides released during cellulose degradation into the cell for catabolism remain unclear. Among its 23 ATP-binding cassette (ABC) sugar transporters, we identified a conserved ABC transporter locus (*athe_0595-0598*) encoding two extracellular binding proteins: Athe_0597 and Athe_0598. Biophysical analyses using differential scanning calorimetry and isothermal titration calorimetry revealed that Athe_0597 binds cello-oligosaccharides of varying lengths (G2-G5), while Athe_0598 is specific to cellobiose (G2). Ligand docking simulations supported these findings and shed light on the subsite configuration of these substrate-binding proteins (SBPs). To assess its physiological importance, we genetically deleted this transporter locus in *A. bescii* strain HTAB187, which grew poorly on cellobiose and did not grow on cellulose. Comparison of growth with a *msmK* deletion strain that cannot consume oligosaccharides showed that HTAB187 retains growth on non-cello-oligosaccharides and monosaccharides. Taken together, these results integrate biophysical characterization, structural modeling, and genetic perturbation to elucidate how *A. bescii* transports cello-oligosaccharides released from cellulose, providing mechanistic insight relevant to consolidated bioprocessing applications.

**IMPORTANCE:**

*Anaerocellum bescii* is the most thermophilic lignocellulolytic bacterium known and holds potential for bioprocessing lignocellulosic biomass into renewable fuels. Its diverse ATP-binding cassette (ABC) sugar transporters make it a valuable model for studying thermophilic sugar uptake. Here, we identify a single ABC transporter with two substrate-binding proteins (Athe_0597 and Athe_0598) responsible for cello-oligosaccharide uptake. Genetic deletion of this transporter locus impaired growth on cellobiose and eliminated growth on cellulose. This is the first genetic manipulation in *A. bescii* to modulate transport of a specific sugar. We also characterize the substrate specificity of the extracytoplasmic binding proteins associated with the locus. One binds various cellodextrins (G2-G5), while the other specifically binds cellobiose (G2). Molecular modeling depicts how each oligosaccharide is docked within the binding pocket of these proteins. Understanding the mechanism of cello-oligosaccharide uptake by *A. bescii* expands opportunities for its metabolic engineering and furthers our understanding of its carbohydrate utilization systems.

## INTRODUCTION

Bio-based fuels and chemicals derived from plant biomass are an attractive and renewable replacement for petroleum-based supply chains (1, 2). However, for decades, technological progress has been stalled by the physical and chemical recalcitrance of lignocellulose, impeding deconstruction and conversion at the large scales needed to compete with comparatively cheaper petroleum (3, 4). Lignocellulolytic thermophiles, with their native ability to overcome lignocellulose recalcitrance, have therefore garnered significant interest, as reflected in recent advances in their genetic and metabolic engineering (5–7). Fully realizing their potential will require a more comprehensive understanding of their physiology and of their native ability to utilize complex carbohydrate substrates (8, 9).

*Anaerocellum bescii* is the most thermophilic, lignocellulolytic bacterium known, with an optimal growth temperature of 75°C (10–12). It possesses vital attributes for consolidated bioprocessing of lignocellulosic feedstocks: efficiently degrading cellulose and hemicellulose into oligosaccharides while simultaneously metabolizing pentose and hexose sugars (13). Multiple genetic tools, including methods for chromosomal modification, an antibiotic-based selection marker, and a xylose-inducible promoter, have been developed over the past decade (14, 15). These tools have enabled metabolic engineering in *A. bescii* to produce a range of valuable products, including ethanol, acetone, and 2,3-butanediol (2,3-BDO), at elevated yields (6, 16–18).

*A. bescii* naturally secretes a suite of multi-domain carbohydrate-active enzymes (CAZymes) for plant biomass deconstruction (19). Its unique glucan degradation locus (GDL) encodes six multi-domain glycoside hydrolases (GHs) with catalytic activity on a broad range of plant biomass substrates, including cellulose, xylans, and mannans (20, 21). Among these, CelA (Athe_1867)*,* CelC (Athe_1857), and CelE (Athe_1865) show particularly strong synergistic activity on cellulose (21). CelA is among the most effective bacterial cellulases known and one of the most abundant proteins in the *A. bescii* secretome (22, 23). It contains an N-terminal GH9 endoglucanase domain, followed by three carbohydrate-binding module family 3 (CBM3) domains for binding cellulose, and a C-terminal GH48 domain exhibiting exo-β-1,4-glucanase activity. Synergistic action between the GH9 and GH48 enables CelA to excavate pits in cellulose microfibrils, exposing interior fibrils, with the GH9 generating new chain ends for cleavage by the GH48, primarily into cellobiose (22–24). Inter-enzyme synergy of CelA with other GDL CAZymes, particularly the GH10-GH48 domains in CelC and GH9-GH5 domains in CelE, further enhances cellulose degradation (21–23). Beyond the GDL, the *A. bescii* secretome also includes auxiliary catalytic and non-catalytic proteins that aid in the deconstruction and uptake of plant biomass polysaccharides (21, 25–27). But despite considerable advances in our biochemical understanding of *A. bescii* plant biomass deconstruction, comparatively little is known about how this bacterium imports the released oligosaccharides into the cell for metabolism (8).

To facilitate carbohydrate uptake, *A. bescii* utilizes ATP-binding cassette (ABC) sugar transporters that hydrolyze two molecules of ATP for the translocation of one sugar molecule into the cytoplasm (13, 28, 29). Twenty-three such ABC transporters have been annotated in the bacterium (13, 30). In addition, the genome encodes a phosphotransferase system (PTS) transporter, which is thought to be fructose specific (30). ABC transporters comprise a hydrophobic transmembrane protein domain, a cytoplasmic ATPase domain, and an extracytoplasmic substrate-binding domain (28, 29). This substrate-binding domain, also referred to as a substrate-binding protein (SBP), is responsible for binding extracellular sugars and delivering them to the transmembrane domain and, consequently, is indicative of the transporter’s carbohydrate specificity (8, 31, 32). There is also genetic evidence to suggest that MsmK, a promiscuous ATPase encoded by *athe_1803*, is responsible for powering all oligosaccharide ABC sugar transporters in *A. bescii* (13). Though a few transcriptomic studies have attempted to predict the specificity of the ABC sugar transporters in *A. bescii*, only those for the maltodextrin system have been experimentally validated thus far (13, 30, 33). No studies have examined how *A. bescii* uptakes oligosaccharides derived from cellulose, a major growth substrate (25).

In this study, we report the first biochemical investigation of cello-oligosaccharide transport in *A. bescii*. We show that two SBPs in *A. bescii*—Athe_0597 and Athe_0598—are specific to cello-oligosaccharides. We demonstrate that these proteins bind cello-oligosaccharides at characteristically high binding affinities, with *in silico* models that structurally elucidate how these sugars are bound. We also genetically deleted both the substrate-binding and transmembrane domains comprising the cello-oligosaccharide ABC transporter locus (*athe_0595–0598*) in *A. bescii*, which resulted in reduced growth on the disaccharide cellobiose and complete loss of growth on cellulose, demonstrating the critical role of this locus in cellulose utilization. These insights reveal essential features of sugar transport in lignocellulolytic *A. bescii* and open doors to engineering strains with novel modes of plant biomass utilization.

## RESULTS

### Identification of the cello-oligosaccharide ABC transport system in *A. bescii*

**Fig 1:**
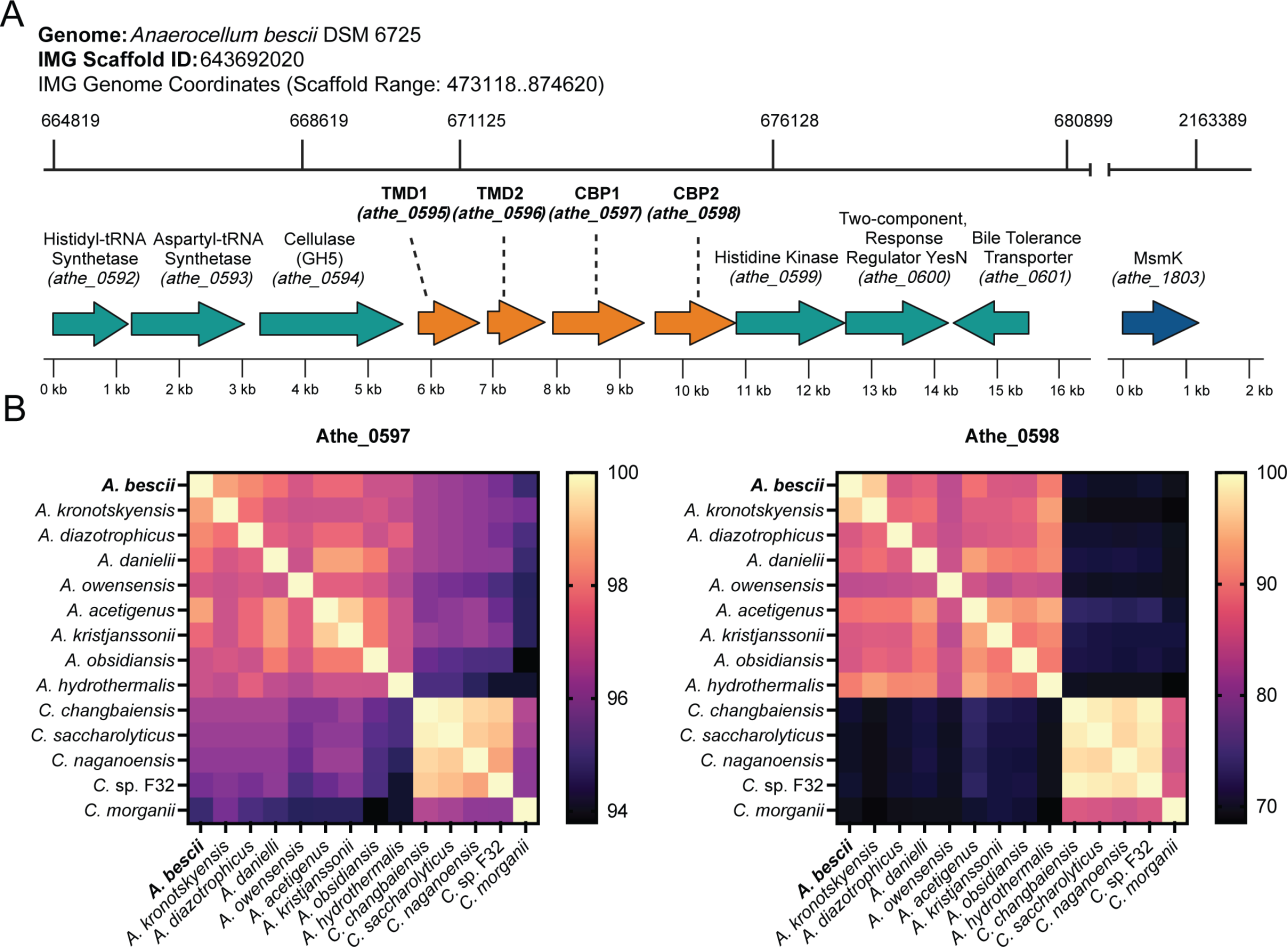
(**A**) Genomic organization and coordinates of the *Anaerocellum bescii* cello-oligosaccharide ATP-binding cassette (ABC) transporter and its neighboring genes. The promiscuous ATPase MsmK, encoded by *athe_1803,* is not a neighboring gene but is also shown due to its involvement in ABC transport of cello-oligosaccharides. TMD = transmembrane domain; CBP = cello-oligosaccharide-binding protein. (**B**) BLAST analysis of protein sequence conservation of cello-oligosaccharide binding proteins Athe_0597 and Athe_0598 across the *Anaerocellum* and *Caldicellulosiruptor* genus.

*A. bescii* contains a gene cluster that encodes an ABC transporter (*athe_0595–0598*) predicted to mediate cello-oligosaccharide transport (Fig. 1A) based on transcriptomic work by VanFossen et al. (2009) in *Caldicellulosiruptor saccharolyticus* and Rodionov et al. (2021) in *A. bescii* (13, 30). Rodionov et al. (2021) further predicted that *athe_0597* and *athe_0598* encode for ABC SBPs with specificity toward oligosaccharides (13, 28). Notably, their expression was upregulated when *A. bescii* was grown on cellulose (13). ABC transporters with multiple SBPs have been documented in other bacteria (34, 35). However, to date, both transporter substrate predictions from Vanfossen et al. (2009) and Rodionov et al. (2021) have not been experimentally validated (13, 30).

The genomic context of the *athe_0595–0598* locus suggests that this ABC transporter plays a role in cello-oligosaccharide uptake. Both genes are co-localized with *athe_0594,* which encodes a cell surface localized GH5-CBM28 endoglucanase with activity on β-glucans from barley, lichenan, and cellulose (20, 36, 37). While Athe_0594 potentially generates cello-oligosaccharide products from endo activity on cellulose, it is thought to play a more minor role in cellulose hydrolysis compared to the GDL enzymes, particularly CelA (20, 21). The *athe_0594–0598* locus was found to be upregulated over 10-fold in *A. bescii* strains grown on cellulose and cellobiose growth substrates (13). Because no ATPase gene is encoded nearby and deletion of the promiscuous *msmK* ATPase (*athe_1803*) disrupts growth on oligosaccharides, including cello-oligosaccharides, this indicates that the Athe_0595*–*0598 cello-oligosaccharide ABC transporter is powered by MsmK (13).

The *athe_0595–0598* ABC transporter gene cluster appears widely conserved across the *Anaerocellum* and *Caldicellulosiruptor* genera, though perhaps to a lesser extent in the latter (the *Anaerocellum* genus was recently split from the *Caldicellulosiruptor* genus) (Fig. 1B) (12). All *Anaerocellum* and *Caldicellulosiruptor* species possess genes encoding the heterodimeric transmembrane domain, as well as genes encoding for the two separate SBPs. The SBP Athe_0597 appears much more highly conserved across both genera. Its *C. morganii* homolog, being the most distinct in amino acid sequence, still retains a relatively high amino acid sequence identity of 94.8% compared to Athe_0597. Conversely, Athe_0598 homologs exhibit higher amino acid sequence variability, particularly among *Caldicellulosiruptor* species as in the cases of *C. saccharolyticus*, *C. changbaiensis*, *C. naganoensis*, *C.* sp. F32 and *C. morganii*. Yet even within the *Anaerocellum* genus, *A. owensensis* has the lowest amino acid sequence identity at 83.6%.

### Biophysical determination of cello-oligosaccharide specificity

Differential scanning calorimetry (DSC) is useful for screening the carbohydrate specificity of highly thermostable SBPs (33, 38). DSC can reach temperatures as high as 130°C, necessary for denaturing *A. bescii* SBPs that typically possess native melting temperatures above 92°C, the limit of traditional circular dichroism instruments (33). In DSC, a constant rate of heating raises the heat capacity of the protein until it fully denatures. The temperature corresponding to the maximum heat capacity of the protein, implying complete denaturation, is the native melting temperature *T*_*m*_ of the SBP in its *apo* state. When bound to its cognate substrates, however, these SBPs acquire a closed conformation with higher thermal stability—a process referred to as the “Venus flytrap” mechanism (8, 31). The difference in melting temperatures across the *apo* and *holo* states, defined as |Δ*T*_*m*_| = *T*_*m*,holo_ – *T*_*m*,apo_, denotes the sugar specificity of a given SBP. Sugars with higher affinity will yield greater Δ*T*_*m*_ values (33).

**Fig 2:**
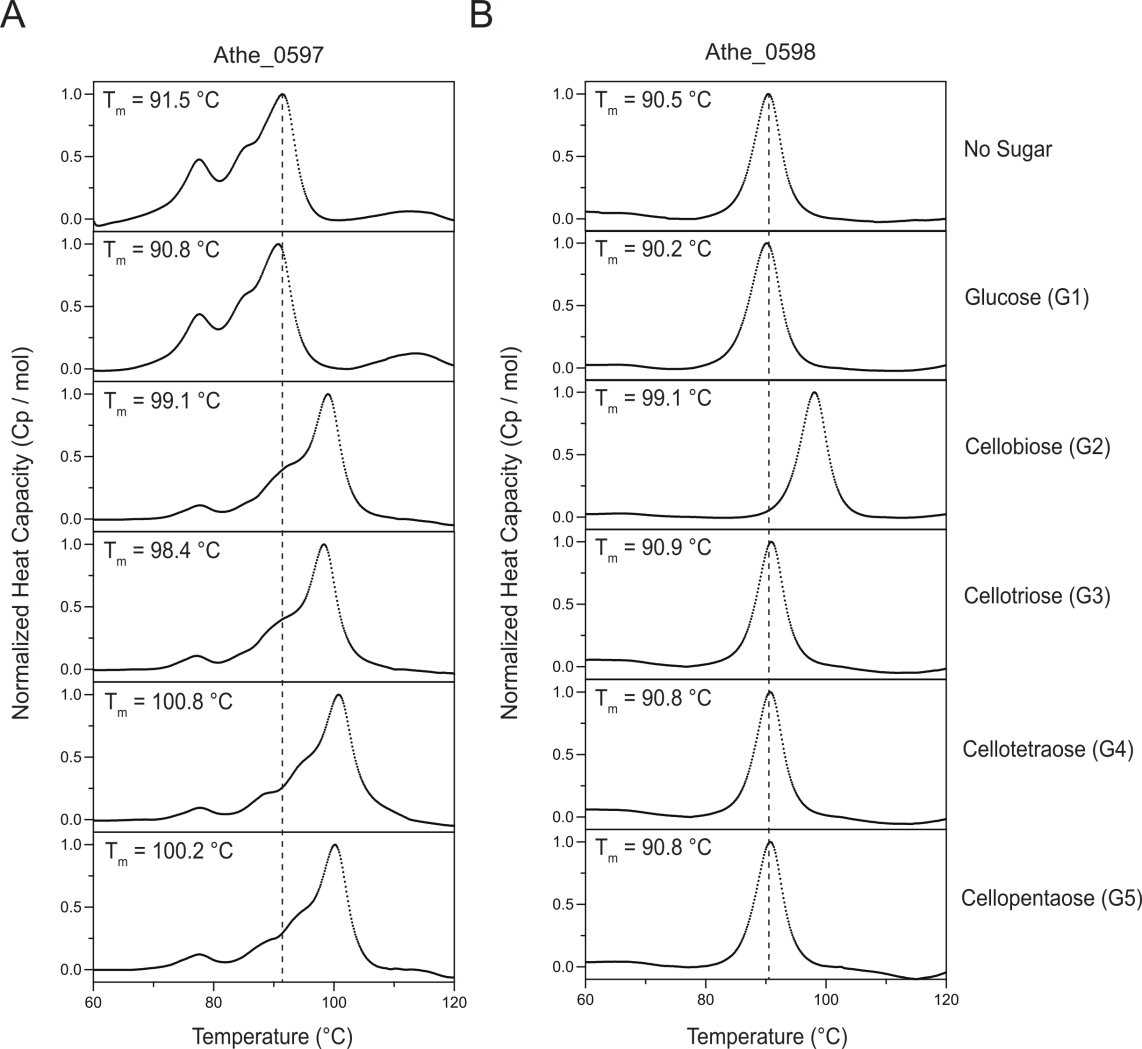
Normalized differential scanning calorimetry (DSC) screens of (**A**) Athe_0597 and (**B**) Athe_0598, mixed with glucose and cello-oligosaccharides of different lengths: cellobiose (G2), cellotriose (G3), cellotetraose (G4), and cellopentaose (G5). Apo melting temperatures for Athe_0597 and Athe_0598 are denoted by a vertical dashed line, respectively. Athe_0597 is shown to bind all tested cello-oligosaccharides from cellobiose to cellopentaose, whereas Athe_0598 only binds cellobiose. All DSC screens were performed at a temperature range of 60–130°C, in 50 mM HEPES and 300 mM NaCl, pH 7.0 buffer.

DSC melting curves showed that Athe_0597 has a strong affinity for cello-oligosaccharide substrates (G2 – G5). In its *apo* state, Athe_0597 exhibited a melting curve with a lower peak at *T*_*m*,1_ = 77.6°C and a higher peak at *T*_*m*,2_ = 91.5°C (Fig. 2). The higher peak at *T*_*m*,2_ = 91.5°C is the presumptive melting temperature, but it is possible that in the absence of a bound ligand, a subunit begins denaturing at *T*_*m*,1_ = 77.6°C (Table 1). The melting curve of Athe_0597 resolves as a single peak in the presence of cognate sugar ligands: cellobiose (G2), cellotriose (G3), cellotetraose (G4), and cellopentaose (G5) with increased melting temperatures of 98.4°C to 100.8°C (Table 1). Curiously, melting temperature shifts are relatively similar for all cello-oligosaccharides that lead to substrate binding (Δ*T*_*m*_ = 6.89 - 9.30°C). The rank ordering of melting temperature increases also does not neatly correlate with oligosaccharide size. This contrasts the larger differences in Δ*T*_*m*_ values observed for the binding of *A. bescii* maltodextrin-binding protein Athe_2574 with maltodextrins of various lengths, from maltose (Δ*T*_*m*_ = 5.03°C) to maltoheptaose (Δ*T*_*m*_ >12°C) (Table 1) (33). Athe_0597 also showed no changes in its melting profile relative to its *apo* state in the presence of glucose, demonstrating its lack of affinity for the monosaccharide.

**TABLE 1:** Biophysical and thermodynamic parameters of binding between substrate-binding proteins (SBPs) and cellodextrins of various lengths as determined by DSC and ITC^*a*^

Protein	Sugar	*T*_m_ (°C)	Δ*T*_*m*_ (°C)	*n*	*K*_*d*_ (μM)	*K*_*a*_ (× 10^5^ M^−1^)	Δ*H* (kcal/mol)	*T* × Δ*S* (kcal/mol)	Δ*G* (kcal/mol)
Athe_0597	Glucose	90.79	−0.70	–	–	–	–	–	–
	CelloG2	99.06	7.57	0.95± 0.02	0.228± 0.10	43.86	3.33± 0.13	12.4	−9.07
	CelloG3	98.38	6.89	1.13± 0.01	0.942± 0.12	10.62	3.94± 0.08	12.2	−8.22
	CelloG4	100.79	9.30	0.95± 0.03	1.97± 0.52	5.08	6.04± 0.32	13.8	−7.79
	CelloG5	100.21	8.72	1.02± 0.08	1.18± 0.12	8.47	4.61± 0.08	12.7	−8.09
Athe_0598	Glucose	90.17	−0.36	–	–	–	–	–	–
	CelloG2	99.05	8.53	2.13± 0.24	2.84± 0.36	3.52	27.2± 10.9	34.7	−7.57
	CelloG3	90.91	0.39	–	–	–	–	–	–
	CelloG4	90.81	0.28	–	–	–	–	–	–
	CelloG5	90.79	0.26	–	–	–	–	–	–

^*a*^ SBP-sugar combinations denoted with a “–” were not tested via the ITC as DSC measurements indicated either weak or absent binding. All measurements were performed at 25°C.

Athe_0598 showed uniform melting even in its unliganded *apo* state (Fig. 2). Binding to cellobiose (G2) did not appreciably alter the melting curve profile compared to the *apo* form. Athe_0598 only exhibited affinity toward cellobiose as a substrate. Larger cello-oligosaccharide substrates from cellotriose to cellopentaose, as well as the monosaccharide glucose, did not result in any *T*_*m*_ shifts, suggesting that none of these ligands were able to bind Athe_0598 (Fig. 2).

**Fig 3:**
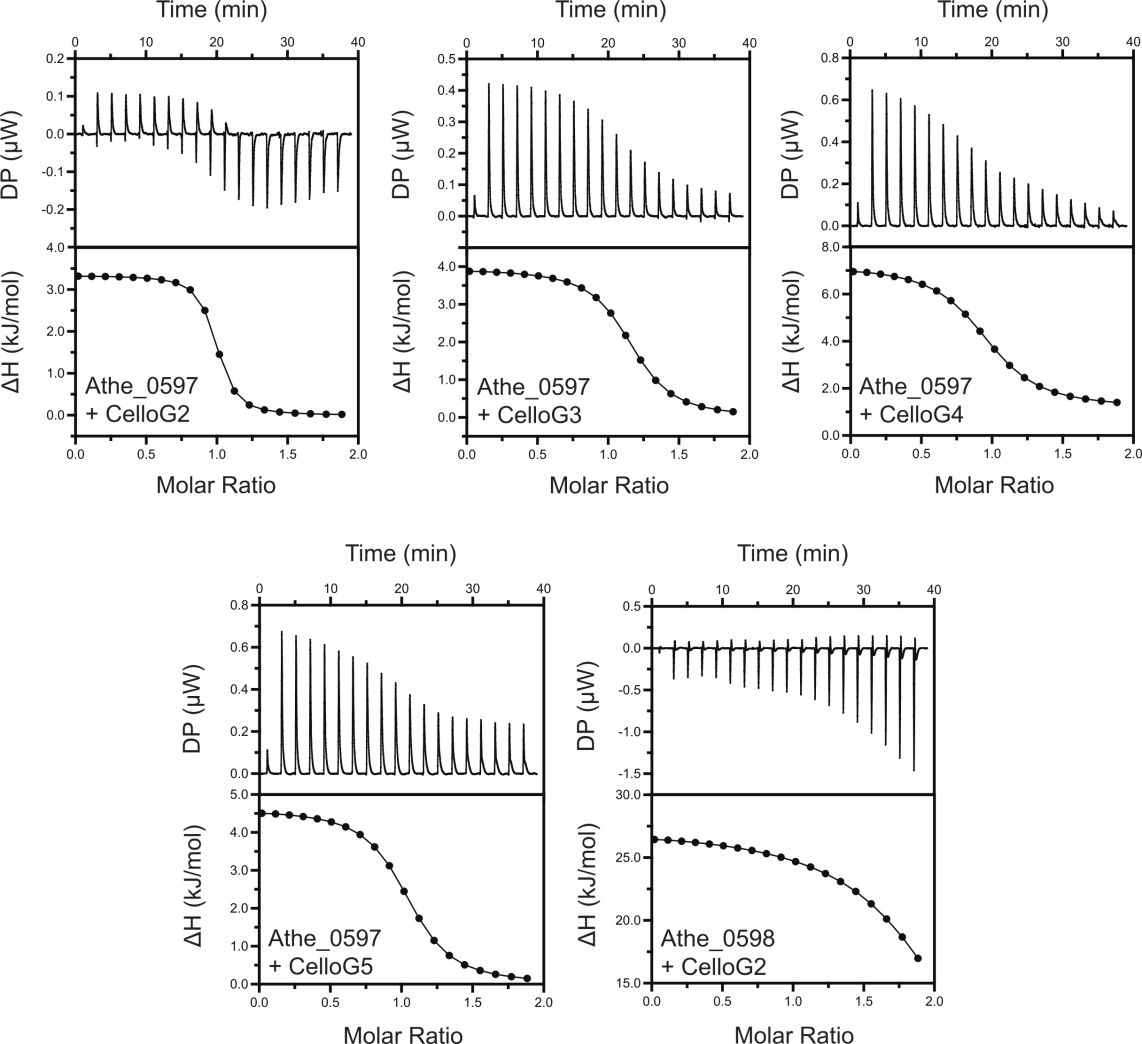
Representative isothermal titration calorimetry (ITC) screens of all SBP and cello-oligosaccharide pairings that were successful from DSC (Fig. 2). These combinations include Athe_0597 with cellobiose (G2), cellotriose (G3), cellotetraose (G4), and cellopentaose (G5), as well as Athe_0598 with cellobiose (G2). Both raw isothermal titration curves and integrated binding isotherms are shown for each SBP and cello-oligosaccharide pairing.

Next, ITC experiments were performed to probe the binding interactions between the two SBPs and their cognate cello-oligosaccharide ligands. Figure 3 illustrates the binding isotherms and isothermal titration calorimetry (ITC) curves for all sugar–protein pairs that were found to bind according to the DSC, with key measurements including dissociation constants *K*_*d*_ and stoichiometry (*n*) summarized in Table 1. ITC data reinforced the results from the DSC, showing that Athe_0597 indeed binds cellobiose, cellotriose, cellotetraose, and cellopentaose with micromolar dissociation constants typical of high-affinity substrates in ABC sugar transporter systems (39, 40). Closer inspection of the association constant *K*_*a*_ values reveals that Athe_0597 has the highest affinity for cellobiose (Table 1). The *K*_*a*_ for cellobiose is one order of magnitude higher than for all other cello-oligosaccharides, including cellotriose after taking into account the observed error (Table 1). The endothermic nature of binding between Athe_0597 and its cello-oligosaccharides is consistent with other known cello-oligosaccharide-binding proteins (39, 40). The binding of both Athe_0597 and Athe_0598 to cello-oligosaccharide substrates also appears to be consistently entropically driven, with *T*Δ*S* values significantly exceeding Δ*H* values in all measured SBP and cello-oligosaccharide combinations (Table 1).

### Docking simulations elucidate structural context to cello-oligosaccharide binding

**Fig 4:**
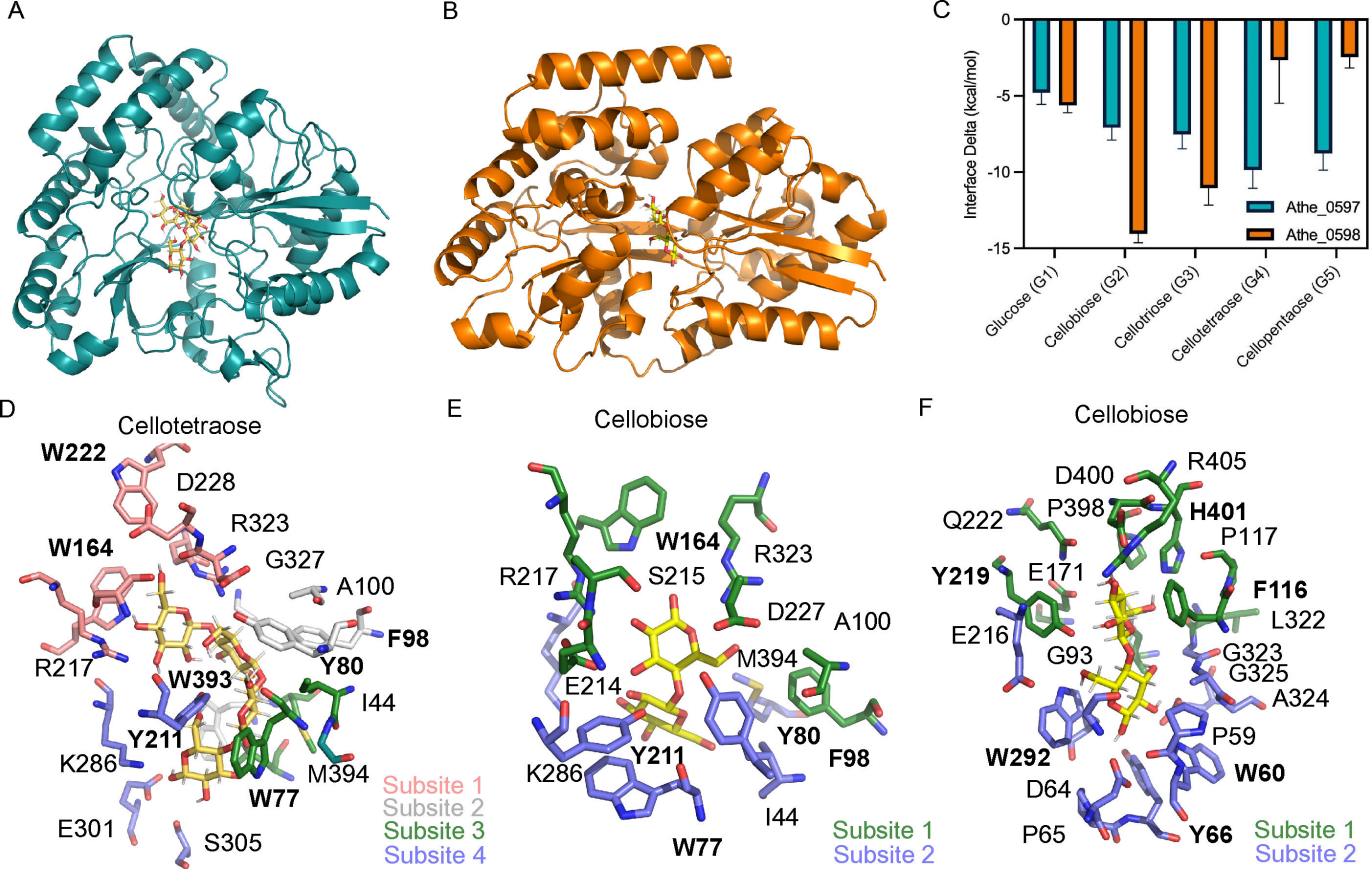
(**A**) Computational model of Athe_0597 as visualized on PyMol. (**B**) Computational model of Athe_0598 as visualized on PyMol. (**C**) Interface energy deltas, as a proxy for free energies of binding, are shown for each simulated combination of cello-oligosaccharide and SBP. The rank-order affinity of Athe_0597 and Athe_0598 with their respective cello-oligosaccharide substrates is aligned with experimental results. (**D**) Binding of the highest affinity ligand to Athe_0597, cellotetraose (G4), is mediated by multiple residues across four subsites in its binding pocket. (**E**) Only subsites 3 and 4 are used to coordinate binding from Athe_0597 to its cognate disaccharide ligand cellobiose (G2). (**F**) Multiple residues in the binding pocket of Athe_0598 mediate the two subsites that coordinate its primary cognate ligand, cellobiose (G2). Residues with aromatic rings are written in bold.

Computational models of protein-carbohydrate interactions showed that Athe_0597 can bind cellobiose, cellotriose, cellotetraose, and cellopentaose, while Athe_0598 primarily favors binding to cellobiose (Fig. 4C; Table S1). These conclusions largely match results obtained through experiments. The modeled Athe_0598 incorrectly retains some affinity for cellotriose, as the closest-match homologs with crystal structures used as templates for the model have affinity for larger oligosaccharides (41). Simulations revealed that each SBP possesses a ligand-binding pocket composed of subsites, where glucosyl monomers can be docked (Fig. 4). Favorable binding free energies are driven by entropic contributions, such as the displacement of water molecules from the binding pocket, and by the formation of specific enthalpic interactions between each glucosyl monomer and the protein at each subsite.

The rank ordering for substrate-binding affinity, as measured by ligand docking for Athe_0597, aligns with the rank ordering indicated by DSC, where cellotetraose is the most favored substrate (Fig. 4C). The non-reducing end of the cellotetraose is bound by subsite 1, with hydrogen bonds between R217 and R323. An internal glucosyl monomer 2 occupies subsite 2 and forms a hydrogen bond with Y80, while a third monomer occupies subsite 3. The reducing end of cellotetraose is bound by subsite 4 through hydrogen bonds between K286, E301, and S305. As shown through ITC, entropic contributions primarily drive substrate affinity (Table 1). Each subsite contains hydrophobic amino acids: W222 and G327 in subsite 1; A100, F98, and W393 in subsite 2; I44, M394, and W77 in subsite 3; and Y211 in subsite 4 (Fig. 4D). When cellobiose is docked in the binding pocket, the disaccharide is stabilized by interactions and residues similar to those that make up subsites 3 and 4 (Fig. 4E).

In the Athe_0598 case, there were fewer available subsites for forming enthalpic interactions and fewer hydrophobic residues in the binding pocket contributing to desolvation entropy. Athe_0598 had the most favorable binding interface energy with the disaccharide cellobiose, as only two subsites are available (Fig. 4F). The non-reducing end of cellobiose is bound by subsite 1, where D400, H401, and E171 form hydrogen bonds and I169, L322, P117, P398, and F116 form hydrophobic contacts. The reducing end occupies subsite 2, where E167, Y219, and D64 form hydrogen bonds and W60, Y66, and W292 form hydrophobic contacts (Fig. 4F). Moreover, the selective binding of cellobiose to Athe_0598 may be due to the limited availability of subsites that could form possible enthalpic interactions, or the lack of available hydrophobic patches for entropic desolvation.

Broadly, our computational analysis suggests a possible mechanism for the entropically driven binding of cello-oligosaccharides to Athe_0597 and Athe_0598. This binding mode is driven by the entropically favorable liberation of solvent molecules when the docked sugar interacts with the hydrophobic aromatic residues located in the hinge region of the proteins. Entropically driven binding is consistent with previous studies showing that protein interactions often include a significant hydrophobic component (42). In line with our findings, our docking models—and those from other studies—indicate that aromatic residues form a hydrophobic cleft around the sugar ring faces.

### Genetic deletion of the cello-oligosaccharide ABC transporter locus (*athe_0595–0598*) disrupts growth on cellobiose and cellulose

To probe its function in carbohydrate assimilation, the cello-oligosaccharide ABC transporter locus (*athe_0595–0598*) was deleted *in vivo* via homologous recombination from the uracil biosynthesis-deficient parent strain MACB1018 (Δ*pyrE*) (15). The cello-oligosaccharide transporter deletion strain HTAB187 (Δ*pyrE* Δ*athe_0595–0598)* was generated using maltose as the carbon source following transformations.

**Fig 5:**
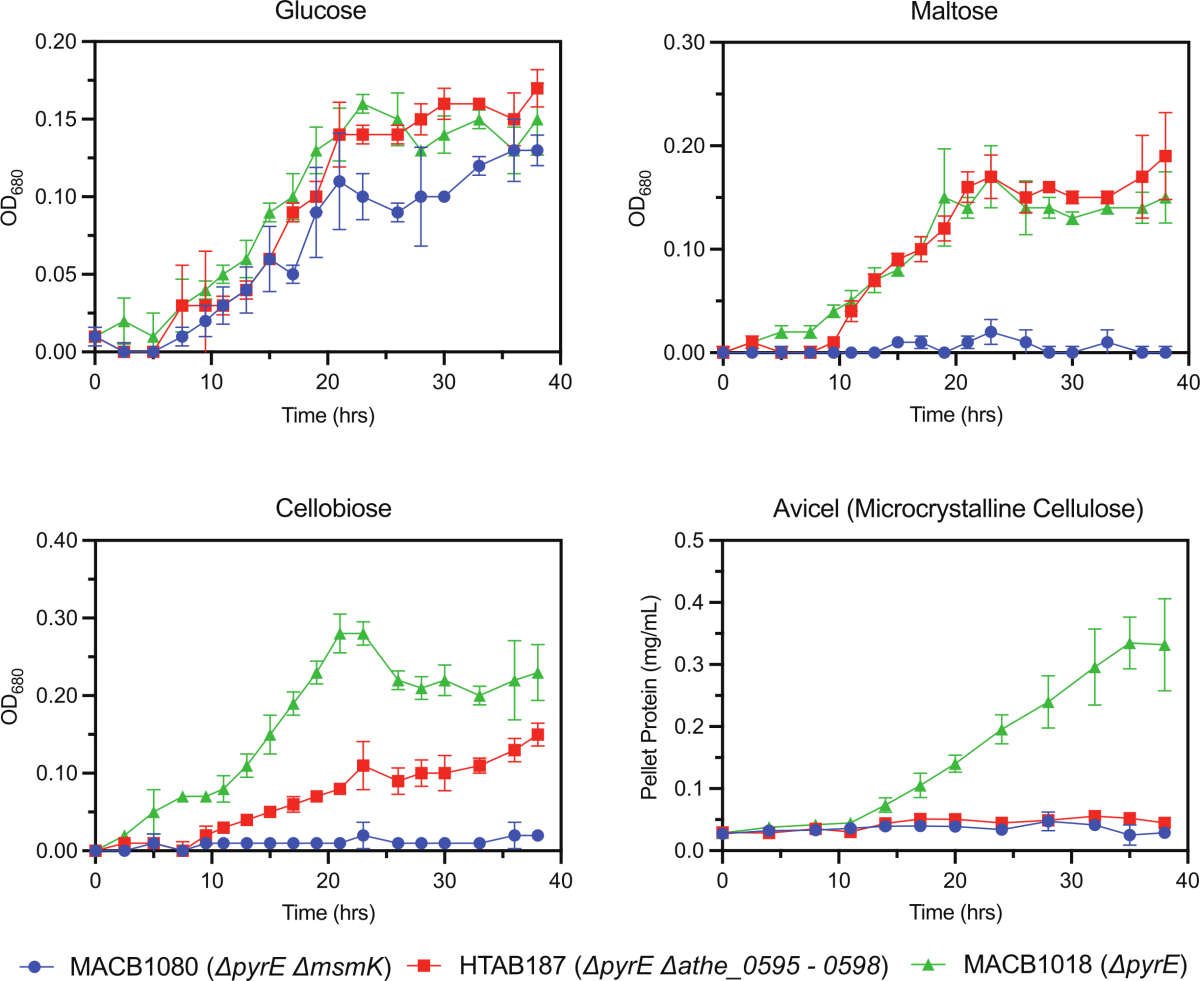
Growth curves of axenic, engineered *A. bescii* strains MACB1018 (green triangles), MACB1080 (blue circles), and HTAB187 (red squares) on carbohydrate substrates glucose, maltose, cellobiose, and Avicel (microcrystalline cellulose). All growth curves were performed in biological triplicate. For growth curves on glucose, maltose, and cellobiose, each data point represents the mean optical density at 680 nm (OD_680_) value. The growth curve on Avicel was based on quantification of pellet protein as previously described (43). Error bars denote the standard deviation.

We conducted growth curves using strain HTAB187 on individual carbon sources, including glucose, maltose, cellobiose, and Avicel (microcrystalline cellulose) (Fig. 5). We also monitored growth curves for the parent strain MACB1018 (Δ*pyrE*) and the MsmK deletion strain MACB1080 (Δ*pyrE* Δ*msmK*). For the latter, we sought to investigate whether a single cello-oligosaccharide transporter deletion results in growth disruption similar to that observed with the inactivation of all oligosaccharide transporters in *A. bescii* (13)*.* To ensure complete inactivation of the transporter, we deleted the entire *athe_0595–0598* locus rather than individual components, as has been done in *Clostridium thermocellum* transporter studies (39).

Unsurprisingly, all three strains demonstrated similar growth behavior on glucose: HTAB187 grows similarly to MACB1018, while MACB1080 exhibits a slightly lower growth rate compared to MACB1018 during the exponential phase (Fig. 5), consistent with prior investigations (13). HTAB187 also exhibited no deviations in growth behavior compared to MACB1018 on maltose, whereas growth on this disaccharide is impaired for MACB1080 due to inactivation of its maltodextrin transporters through loss of ATPase MsmK. On cellobiose, however, all three strains displayed distinct growth behavior. Growth on cellobiose was clearly impaired for HTAB187 compared to MACB1018, although the defect was not severe enough to completely prevent growth. Surprisingly, MACB1080 showed no growth whatsoever on cellobiose in contrast with literature results that suggest minimal growth on the disaccharide (13). And despite some growth on cellobiose, HTAB187 appears to be entirely incapable of growth on cellulose. MACB1080 also showed no growth on cellulose, consistent with previous reports (13). Visual inspection of the turbidity of cultures from all three strains on cellulose suggested that only MACB1018 was thriving (Fig. S1). Broadly, deletion of the *athe_0595–0598* cello-oligosaccharide transporter locus results in (i) significant, but partial, impairment of growth on cellobiose, and (ii) elimination of growth on cellulose, without apparent defects in the uptake of other glucose-based substrates, such as glucose or maltose. Although it is possible that cellobiose can be extracellularly hydrolyzed into glucose, which HTAB187 can uptake, it is more likely that low-affinity transport through another ABC transporter explains the limited growth on cellobiose. If the former was the case, we would have expected partial MACB1080 growth on cellobiose given that it can still grow on glucose. Overall, these results indicate that Athe_0595–0598 is the major cello-oligosaccharide transporter in *A. bescii.*

## DISCUSSION

Here, using biophysics, structural modeling, and genetic knockouts, we show that Athe_0595–0598 is the principal ABC transporter that enables *A. bescii* to utilize cello-oligosaccharides. Through DSC, we screened the substrate specificity of SBPs Athe_0597 and Athe_0598. We further characterized and quantified their substrate-binding thermodynamics using ITC, showing that both SBPs bind to cello-oligosaccharides with dissociation constants in the μM range, characteristic of high-affinity ABC sugar transporters (33, 40, 44). Using sequence and structural alignment to known thermophilic homologs, as well as molecular modeling, we further elucidated how these carbohydrate ligands are docked within their respective SBP binding pockets. Finally, we complemented *in vitro* and *in silico* characterization with knockout of the *athe_0595-0598* locus *in vivo* and subsequently studied changes to growth behavior on multiple carbohydrate sources. We showed that deletion of this transporter locus impaired growth on cellobiose and eliminated growth on cellulose, illustrating its critical role in cello-oligosaccharide and cellulose utilization.

Both ITC and DSC corroborated that Athe_0597 and Athe_0598 bind cello-oligosaccharide substrates with *K*_*d*_ values in the μM range (Table 1). While Athe_0597 binds to cello-oligosaccharides of various lengths, from cellobiose to cellopentaose with similar *T*_*m*_ shifts, Athe_0598 only binds to cellobiose. Although ITC and DSC largely agree that cello-oligosaccharides of lengths G2–G5 induce high-affinity binding with Athe_0597, differences in thermal stabilization and *K*_*d*_ values are not as tightly correlated. We found a decreasing rank order between *T*_*m*_ shift and oligosaccharide length for Athe_0597 (G4 > G5 > G2 > G3), whereas the rank order for decreasing binding affinity (decreasing *K_a_*) follows the trend of G2 > G3 > G5 > G4 (Table 1). In the context of the maltodextrin transport system, which includes two orthogonal SBPs, Athe_0598 could be considered analogous to Athe_2310 as the SBP with higher specificity toward shorter oligosaccharides (33). However, unlike the maltodextrin system where one SBP does not display a significant affinity toward the disaccharide maltose, both Athe_0597 and Athe_0598 bind cellobiose as a cognate ligand. Previously, Yokoyama et al. (45) proposed Athe_0597 as a secreted SBP meant for plant cell wall adhesion (45). Our biophysical measurements demonstrated substrate specificities consistent with those reported in their study. However, our genetic and biochemical evidence indicates that Athe_0597 is not simply a secreted adhesin, but rather the major substrate-binding component of the Athe_0595-0598 ABC transporter system mediating cello-oligosaccharide uptake.

The Athe_0597 and Athe_0598 SBPs are highly conserved in the genera *Anaerocellum* and *Caldicellulosiruptor*, indicating their likely importance (Fig. 1B). Among them, Athe_0597 is more broadly conserved across *Caldicellulosiruptor* species compared to Athe_0598. Athe_0597 is also multi-functional, binding an array of cello-oligosaccharides while binding cellobiose at association constants comparable to those of Athe_0598. This suggests that Athe_0597 plays the more important role in cello-oligosaccharide assimilation. Although all *Anaerocellum* and *Caldicellulosiruptor* species are hemicellulolytic, and most are cellulolytic, some *Caldicellulosiruptor* species, such as *C. kristjanssonii*, lack a GDL and are non-cellulolytic (19). Even in non-cellulolytic species, retaining the cello-oligosaccharide transporter locus may confer a metabolic advantage by enabling the scavenging of cello-oligosaccharides generated by neighboring cellulolytic species in their native hot springs.

**Fig 6:**
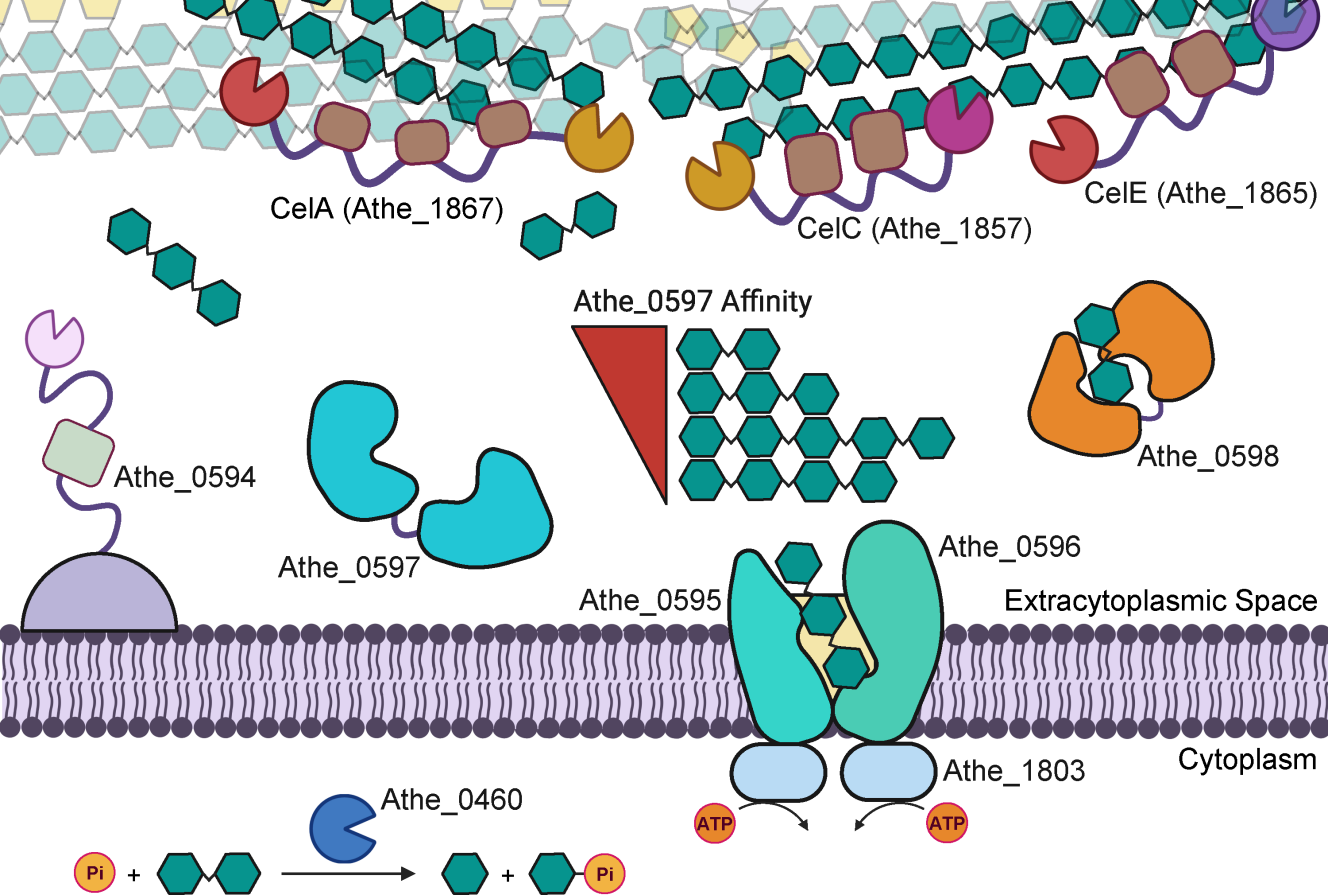
A proposed mechanism of cellulose deconstruction and transport in *Anaerocellum bescii.* In the extracellular space, secreted GDL CAZymes CelA, CelC, and CelE work in concert to deconstruct cellulose. Surface layer associated Athe_0594 also potentially supports cellulose deconstruction through endoglucanase activity that generates cello-oligosaccharides. The released cello-oligosaccharides are first captured by ABC cellodextrin-binding proteins Athe_0597 (with affinity to cello-oligosaccharides of various lengths, ranked in order of decreasing K_a_ as determined by ITC in Table 1) and Athe_0598 (with affinity only to cellobiose), and then delivered to the Athe_0595–0596 permease. Cellodextrin translocation across the membrane is coupled to ATP hydrolysis by the MsmK (Athe_1803) nucleotide-binding domain. Within the cytoplasm, cellobiose phosphorylases, such as those encoded by Athe_0460, phosphorylate cellobiose into glucose and glucose-1-phosphate.

Previous investigations have demonstrated that purified GDL enzyme cocktails, or even the concentrated native secretome itself, are not as effective as a live *A. bescii* culture in solubilizing cellulose, underscoring the importance of simultaneous uptake of cello-oligosaccharides to remove end-product inhibition and maximize cellulose deconstruction (21). Based on these results, we propose a model describing cellulose deconstruction by the coordinated action of GDL CAZymes and the transport of released cello-oligosaccharides by the Athe_0595–0598 transporter (Fig. 6). As Athe_0597 binds cello-oligosaccharides of lengths G2–G5 yet displays the highest affinity for cellobiose (G2), and Athe_0598 binds strictly to cellobiose, cellobiose generated by exoglucanases (e.g., GH48 domains in CelA and CelC) likely predominates among imported sugars over larger cello-oligosaccharides produced by endoglucanase activity (e.g., GH9, GH5).

Transporter selectivity carries important bioenergetic consequences. Multiple studies have suggested that the bioenergetic benefit of transporting higher-order oligosaccharide substrates is vital for offsetting the significant metabolic costs of expressing a large array of extracellular CAZymes for cellulose deconstruction (46, 47). The cost of translocating one sugar molecule across the membrane remains fixed at two ATP molecules regardless of sugar size. An affinity for transporting larger sugars implies a more efficient use of resources (46). Cellobiose is a predominant product of cellulase activity and serves as a ligand for both Athe_0597 and Athe_0598, underscoring its central role in substrate uptake strategies of *A. bescii*. Additionally, *A. bescii* carries intracellular cellobiose phosphorylases, encoded by *athe_0459* and *athe_0460*, which cleave cellobiose into glucose and glucose-1-phosphate, thereby enabling an ATP-saving strategy observed in other cellulolytic anaerobes such as *Acetivibrio thermocellus* (basionym *Clostridium thermocellum*) and *Ruminiclostridium cellulolyticum* (13, 46, 48, 49).

We examined how this transporter’s function contributes to growth on cellulose *in vivo*. Both *A. bescii* strains HTAB187 (Δ*pyrE* Δ*athe_0595–0598*) and MACB1080 (Δ*pyrE* Δ*msmK*) failed to grow on microcrystalline cellulose, suggesting that the promiscuous *msmK* (Athe_1803) is the ATPase associated with the *athe_0595–0598* cello-oligosaccharide transporter locus. This result is also consistent with a prior MACB1080 growth study performed on microcrystalline cellulose (13). Interestingly, while this previous study (13) showed that MACB1080 can experience modest growth on cellobiose, we were unable to reproduce this result. We suspect potential variance in inoculation method, or perhaps fructose carryover from the inoculum, could account for the observed growth of MACB1080 in cellobiose-containing media (13, 39).

HTAB187 demonstrated modest growth on cellobiose compared to the MACB1018 parent strain. Because HTAB187 growth on cellobiose reached final OD_680_ levels comparable to growth on glucose, we speculate two possible causes for this phenomenon. First, it is possible that extracellular β-glucosidase activity could hydrolyze cellobiose into glucose, the latter of which strain HTAB187 can import. However, this seems unlikely, as we did not observe growth of MACB1080 under these conditions, which can also grow on glucose. Additionally, no extracellular β-glucosidases dedicated to hydrolyzing cellodextrins to glucose have been identified in the *A. bescii* secretome, and heterologous expression of an extracellular β-glucosidase in *A. bescii* only modestly affected growth on cellulose (50)*.* Alternatively, a more plausible explanation is that *A. bescii* possesses other MsmK-dependent oligosaccharide transporters with some affinity for cellobiose enabling partial growth on the disaccharide. However, because these redundant transporters may not possess as high a binding affinity for cellobiose, or because they are not as highly expressed, the growth rate of HTAB187 on cellobiose remains noticeably impaired.

The lignocellulolytic bacterium *Ruminiclostridium cellulolyticum* also employs an ABC transporter for cellodextrin uptake (51). Within its seven-gene cellulose utilization associated (*cua*) gene cluster, the SBP CuaD, which is specific only to cellobiose, appears to contribute to regulation of transporter expression, as mutation of *cuaD* impaired growth on cellobiose (51). By analogy, it remains possible that in the *athe_0595–0598 locus*, Athe_0598—which only recognizes cellobiose—could similarly regulate carbohydrate utilization in *A. bescii.* However, unlike CuaD, which exhibited significantly higher affinity for cellobiose than the transport-associated SBP CuaA, Athe_0598 showed lower affinity for cellobiose compared to Athe_0597 (Table 1). Cellodextrin transport also plays a regulatory role in *Clostridium thermocellum*, whereby cellobiose uptake directly induces cellulosome expression (39). In the *C. thermocellum* system, initial biophysical studies suggested that four distinct SBPs were capable of binding cello-oligosaccharides, but subsequent genetic analyses demonstrated that only a single transporter is critical for uptake, with the others potentially serving regulatory or accessory functions (39, 40). In contrast, transcriptomic and proteomic analyses suggest that expression of the GDL CAZymes in *A. bescii* is consistent across diverse growth conditions (13, 25, 39). Decoupling the root cause of growth disruption on cellulose in *A. bescii*—potentially by observing simultaneous cellulose deconstruction alongside growth on non-cellodextrin sugars in a panel of additional mutants—is the most logical next step for disentangling transport and regulation in *A. bescii*.

In sum, this study identifies Athe_0595–0598 as the primary ABC transporter responsible for cello-oligosaccharide uptake in *A. bescii.* Our results reveal that Athe_0597 and Athe_0598 exhibit distinct binding preferences, with Athe_0597 recognizing a range of cello-oligosaccharides and Athe_0598 specifically binding cellobiose. Knockout of the *athe_0595–0598* ABC transporter locus in *A. bescii* strain HTAB187 abolished cellulose utilization and severely impaired growth on cellobiose, underscoring its essential role in assimilation of the cello-oligosaccharide substrates derived from lignocellulose*.* Without its ability to grow on cellulose, strain HTAB187 could conceivably be co-cultured with other cellulose-utilizing thermophiles, such as *Clostridium thermocellum*, for consolidated bioprocessing of plant biomass (52). Our findings illuminate core features of carbohydrate utilization and bacterial physiology in *A. bescii*, as well as in relate*d Anaerocellum* and *Caldicellulosiruptor* species, and establish a framework for future metabolic engineering efforts that involve manipulation of sugar transport.

## MATERIALS AND METHODS

### Bacteria and growth conditions

*E. coli* strains, including NEB 5-α (New England Biolabs) and BL21 (DE3) pRosetta2 (EMD Millipore), were plated routinely on Luria-Bertani medium (10 g/L tryptone, 5 g/L yeast extract, 10 g/L NaCl) with 1.5% agar. All plates were supplemented with 50 µg/mL kanamycin for selection. All *E. coli* strains were cultured routinely in Luria-Bertani broth containing an additional 24 g/L yeast extract. All plates and cultures for growing *E. coli* BL21 (DE3) pRosetta2 strains were supplemented with 33 µg/mL chloramphenicol antibiotic. *A. bescii* strains DSMZ 6725, MACB1018, and MACB1080 were provided by the labs of Dr. Robert Kelly (North Carolina State University) and Dr. Michael W. W. Adams (University of Georgia) and cultured on complex media (C516) containing 5 g/L maltose, 0.5 g/L yeast extract, and 40 µM uracil as described previously (15, 53). Late exponential phase *A. bescii* cells were harvested and pelleted by centrifugation at 5,000 × *g* for 20 min. Genomic DNA was extracted from pelleted cells using a Quick-DNA Miniprep kit (Zymo Research) and quantified using a UV-Vis Spectrophotometer Nanodrop (Thermo Scientific).

### Chemicals

The following monosaccharides and oligosaccharides were used in this study: D-maltose monohydrate (>98.0%, Tokyo Chemical Industry), D-fructose monohydrate (>99.0%, Tokyo Chemical Industry), D-glucose (Thermo Scientific), D-cellobiose (>98%, Acros Organics), D-cellotriose (>95%, Neogen Corporation), D-cellotetraose (>90%, Neogen Corporation), D-cellopentaose (>95%, Biosynth), and Avicel PH-101 cellulose resin (Sigma Aldrich).

### Substrate-binding protein cloning

Methods were performed as previously described (33). To recapitulate, genes encoding for the cello-oligosaccharide-binding proteins Athe_0597 and Athe_0598, excluding their respective signal peptides (as predicted by SignalP 5.0), were amplified via PCR from *A. bescii* DSMZ 6725 genomic DNA (54). PCR amplification using Phusion polymerase (New England Biolabs) was performed with primers described in Table S2, according to the manufacturer’s instructions. Athe_0597 was inserted into a pRSF1-b backbone (gift from the Kelly Lab, North Carolina State University), whereas Athe_0598 was inserted into a pCri8a (Addgene Plasmid #61317) backbone. Gene insertion was performed via Gibson Assembly using the NEB HiFi Assembly Master Mix, as detailed in the manufacturer’s instructions. N-terminal hexa-histidine tags were added to each SBP gene to enable protein purification via immobilized metal affinity chromatography (IMAC). The plasmid containing Athe_0597 is hereafter dubbed pHT007, while the plasmid containing Athe_0598 is dubbed pHT017. Each plasmid was transformed to *E. coli* NEB 5-α, with 50 µg/mL kanamycin selection pressure for plasmid maintenance. Plasmids were prepared and purified using the Zymo Research Plasmid Miniprep – Classic Kit (Zymo Research) and sequenced via Plasmidsaurus’ whole-plasmid sequencing service. Sequence confirmed that pHT007 and pHT017 plasmids were each transformed into *E. coli* BL21 (DE3) pRosetta2 strains (EMD Millipore), with double selection markers of 50 µg/mL kanamycin and 33 µg/mL chloramphenicol to maintain both protein expression and pRosetta2 vectors.

### Protein expression and purification

Methods were performed as previously described in (8, 33). *E. coli* BL21 (DE3) pRosetta2 strains transformed with respective pHT007 and pHT017 vectors were grown in 1 L ZYM-5052 auto-induction media supplemented with 50 µg/mL kanamycin and 33 µg/mL chloramphenicol in 2.8 L shake flasks at 37°C and a shaking speed of 250 rpm (55). Cell cultures were harvested after 20 h of overnight growth at 4,500 × *g* for 20 min. Pellets were stored in a −20°C freezer. IMAC Buffer A (20 mM sodium phosphate monobasic, 500 mM sodium chloride, pH 7.4) was used to resuspend cell pellets at a ratio of 10 mL buffer per 1 g of wet cell pellet. An Emulsiflex-C5 High-Pressure Homogenizer (Avestin) was used to lyse resuspended pellets using house airflow maintained at 30 psi. Each cell lysate was subjected to a pressure of approximately 100,000 kPa per pass and a minimum of two full lysis cycles. Homogenized cell lysates were then heat treated at 68°C for 30 min using a water bath. Heat-treated cell lysate was centrifuged in Nalgene Oak Ridge High-Speed PPCO Centrifuge Tubes at 36,000 × *g* for 30 min (Thermo Scientific). The pooled supernatant was filtered through a 0.2 µm PES filter. Using a BioRad NGC 10 FPLC (Bio-Rad), filtered cell extract was applied to a 5 mL HisTrap HP Nickel-Sepharose (Cytiva) column according to the manufacturer’s specifications. Ni-NTA sample load, column wash, and elution samples were selected via inspection of the absorbance curve at 280 nm in the chromatogram and subsequently run on a precast 4–20% Mini-PROTEAN TGX Stain-Free Protein Gel (Bio-Rad), using the Precision Plus Protein Standard (Bio-Rad) as the protein molecular weight ladder. Purified elution fractions with high protein quantity and purity were selected, pooled, concentrated, and buffer-exchanged using a 10 kDa MWCO PES filter 20 mL Spin-X Concentrator (Corning). The final protein storage and characterization buffer is comprised of 50 mM HEPES, 300 mM NaCl, pH 7.0. A bicinchoninic acid (BCA) assay (Thermo Fisher Scientific) was used to quantify pHT007 concentrated to 50 mg/mL and pHT017 concentrated to 10 mg/mL. Both proteins were run on SDS-PAGE to verify protein identity by weight and sample purity (Fig. S2).

### Isothermal titration calorimetry (ITC)

Methods were performed as previously described (33). Briefly, measurements were performed at 25°C using a MicroCal PEAQ Isothermal Titration Calorimeter (Malvern Panalytical) hosted by the Princeton University Biophysics Core Facility. Standard titration experiments were performed using a protein sample volume of 280 µL at a concentration of 50 µM and a sugar ligand volume of 40 µL at a concentration of 500 µM. A cell stir speed of 750 rpm was used throughout the entire titration experiment. The protein in the cell was initially injected with a priming aliquot of 0.2 µL sugar, followed by 19 injections of 2.0 µL each, with 2-minute intervals between injections. A single-site binding model was used to determine integrated heat effects via non-linear regression (Microcal PEAQ-ITC Analysis). Binding dissociation constant and key thermodynamic parameters were determined from the fitted isotherms using the Gibbs free energy form ΔG = -RTln(*K*_*d*_). The dissociation constant *K*_*d*_ was calculated based on the binding isotherm’s slope at the equivalence point, and its inverse was taken as the association rate constant *K*_*a*_. 280 µL of Milli-Q water was used in the reference cell.

### Differential scanning calorimetry (DSC)

Methods were performed as previously described (33). Briefly, the melting curve profiles of hexa-histidine-tagged Athe_0597 and Athe_0598 were determined via DSC using the MicroCal PEAQ-DSC (Malvern Panalytical) hosted at the Princeton University Biophysics Core Facility. Athe_0597 and Athe_0598 were screened against a palette of cello-oligosaccharide substrates: cellobiose (G2), cellotriose (G3), cellotetraose (G4), and cellopentaose (G5). Glucose (G1) was also tested as a negative control. Each protein–ligand mixture contained final concentrations of 2 mg/mL protein and 5 mM sugar, with the same protein storage buffer (50 mM HEPES, 300 mM NaCl, pH 7.0) used as the reference. All scan rates were set at 4°C/min, reaching a maximum temperature of 130°C. Raw data consisted of the heat capacity (*C*_*p*_) plotted as a function of temperature, followed by normalization to each respective run’s maximum heat capacity value, *C*_*p*,max_. *C*_*p*,max_ is defined as *C*_*p*,max_ = *C*_*p*_ (*T* = *T*_*m*_), where *T*_*m*_ is the melting temperature of the protein mixture. Each protein–carbohydrate mixture was ascribed a Δ*T*_*m*_, defined as Δ*T*_*m*_ = *T*_*m*,holo_ – *T*_*m*,apo_, where *T*_*m*,holo_ is the melting temperature of a given protein–sugar mixture and *T*_*m*,apo_ is the melting temperature of the protein in the absence of sugar.

### Construction of *A. bescii Δathe_0595–0598* knockout strain

Starting with the MACB1018 background parent strain (Δ*pyrE*), the *athe_0595-0598* gene cluster was chromosomally deleted via homologous recombination using previously described methods (15) (Fig. S3). The plasmid pKHT017 contained a ~ 1 kb flanking region upstream of the *athe_0595* gene and a ~ 1 kb flanking region downstream of the *athe_0598* gene. Both flanking regions are adjacent to one another in the knockout vector (Fig. S2). Flanking regions were PCR-amplified from *A. bescii* DSMZ 6725 genomic DNA using Phusion polymerase (New England Biolabs) and inserted into a pGL103 backbone, as previously described (15). The knockout vector was transformed into *E. coli* NEB 5-α via heat shock at 42°C, followed by recovery in SOC outgrowth medium (New England Biolabs) and then growth on plates and in cultures supplemented with 50 µg/mL kanamycin and 50 µg/mL apramycin selection pressures for plasmid maintenance. pKHT017 was prepared and purified using the Zymo Research Plasmid Miniprep – Classic kit (Zymo Research) and sequenced via Plasmidsaurus’ whole-plasmid sequencing service, as described above. Sequence confirmed pKHT017 transformants were grown in 250 mL Luria-Bertani medium supplemented with additional 24 g/L yeast extract, as well as 50 µg/mL kanamycin and 33 µg/mL apramycin antibiotics, in 1 L shake flasks at 37°C. Incubator shake speed was set at 250 rpm. Cells were pelleted and processed via a ZymoPURE II Plasmid Purification Maxiprep kit. The pKHT017 plasmid was then methylated *in vitro* with M.CbeI methyltransferase, as previously described (15, 56). Successful methylation was verified by protection against HaeIII restriction endonuclease activity.

Competent MACB1018 cells were prepared in low-osmolarity-defined (LOD) media containing 5 g/L cellobiose and 40 µM uracil, as described previously (15, 53). LOD media and all media hereafter described were degassed and made anaerobic for 15 min using a vacuum pump, with a gaseous headspace comprising 80% (vol/vol) nitrogen and 20% (vol/vol) carbon dioxide gas. Competent cells were electroporated using a Bio-Rad Gene Pulser with 2 µg of methylated pKHT017 plasmid at 2.2 kV, resistance of 400 Ω, and capacitance of 25 µF. One milliliter of recovery media, containing modified DSM 516 media supplemented with 5 g/L of yeast extract and preheated to 70°C, was immediately used to resuspend electroshocked cells (15). After both 60 min and 120 min post-electroporation, 5 mL of electroporated cells in recovery medium were transferred to 50 mL (selective) DSM 516 medium supplemented with 50 µg/mL kanamycin. Cloudy media after 48–72 h of growth at 70°C suggested successful transformations; 1 mL samples of such cultures were passaged into 10 mL fresh selective DSM 516 media containing 50 µg/mL kanamycin. Passaged transformants were then plated in solid, selective modified DSM 516 medium with 1.5% agar, grown for 48–72 h at 70°C under 95% (vol/vol) nitrogen and 5% (vol/vol) hydrogen gas, and single colonies were selected and screened via PCR to confirm knockout vector chromosomal integration adjacent to the *athe_0595-0598* locus (13). A PCR-verified first crossover colony is then plated on solid, modified DSM 516 medium containing 8 mM 5-fluoroorotic acid (5-FOA) and 40 µM uracil. The 5-FOA counter-selection step also marks a permanent change in growth substrate from 5 g/L cellobiose to 5 g/L maltose. Single colonies were picked and screened for complete deletion of the *athe_0595-0598* locus following counter-selection, using colony PCR with primers directly outside the 1 kb upstream and downstream flanking regions present in the pKHT017 vector.

### *A. bescii* growth curves on select carbohydrate substrates

Freezer stocks of MACB1018 (Δ*pyrE*), MACB1080 (Δ*pyrE* Δ*msmK*), and HTAB187 (Δ*pyrE* Δ*athe_0595–0598*) were each thawed and used to inoculate 50 mL of anaerobic, non-selective modified DSM 516 medium containing 40 µM uracil and 5 g/L of fructose in 100 mL serum bottles for overnight growth. Strains were passaged once more into anaerobic, non-selective modified DSM 516 medium containing 40 µM uracil and 5 g/L of fructose. Upon reaching late exponential phase at approximately 16 h of growth at 70°C, cultures were cooled to room temperature. Ten milliliters of cells from each strain was extracted and centrifuged at 14,000 rpm for 2 min. The supernatant was primarily decanted by pouring out the liquid, with residual supernatant removed via pipetting. The pellet was then washed and resuspended in 1 mL of non-selective modified DSM 516 sugar-free medium, and then injected into 10 mL of the same sugar-free medium contained within a 18 × 125 mm anaerobic tube sealed with 20 mm butyl rubber stoppers (Duran Wheaton Kimble), resulting in a sugar-free bacterial culture with final OD_680_ ∼0.10.

One milliliter of the sugar-free bacterial culture was used to inoculate 50 mL of non-selective modified DSM 516 medium containing 40 µM uracil and a single carbohydrate source at 5 g/L, sealed by 20 mm butyl rubber stoppers (Duran Wheaton Kimble). All inoculated cultures reported an initial OD_680_ ∼ 0.00 and were grown at 70°C, without shaking, for 40 h in biological triplicate, as previously described (13). The OD_680_ of growth cultures was measured at intervals of roughly 2.5–6.0 h using the cuvette setting of a UV-Vis Nanodrop Spectrophotometer, with 1× DSM 516 salt solution as the blank. Cell protein content, used as a proxy for cell density under Avicel growth conditions, was quantified using the Bradford method with bovine serum albumin (BSA) protein standards (Pierce Thermo Scientific) in a 96-well plate format, as previously described (13, 39, 43).

### Bioinformatics

Through PDB sequence similarity search, glucan-binding protein orthologs with solved crystal structures were identified using BLAST, with Athe_0597 and Athe_0598 as the query protein sequences (57). For Athe_0597, structures were all from *Streptococcus pneumoniae* (PDB: 5SUO, 5SWA, 5SWB) (41). For Athe_0598, structures included those from *Streptococcus pneumoniae* (PDB: 5SWA), as well as *Caldanaerobius polysaccharolyticus* (PDB: 4G68), *Paenibacillus sp. str. FPU-7* (PDB: 7EHP), and *Thermotoga maritima* (PDB: 2O7I) (41, 58–60). Amino acid sequences of all homologs used in this study are summarized in Table S3. PROMALS3D and ESPript 3.0 visualization software were used to identify similarities in secondary and tertiary protein structures from structure-based sequence alignment of each SBP and their orthologs (61, 62).

### Ligand docking models

ColabFold v1.5.5 supplied with AlphaFold 2 parameters was used to generate the initial models. The best-match homolog was used as the template, with other homologs providing the MSA (63, 64). The “ref2015” score function under the Rosetta *relax* protocol was used to minimize AlphaFold-generated structures. Docking of carbohydrate ligands onto protein binding pockets was performed using the ROSIE server (65, 66). Initial posing of the carbohydrate ligand within the protein binding pocket was based on homology to the corresponding crystal structure. For Athe_0597, glucose was positioned in the binding cleft from crystal structure 5SWI; cellobiose was positioned in the two subsites of 5SWA; cellotriose was positioned in three subsites of 5SWB; cellotetraose was positioned in four subsites of 5SWB; and cellopentaose was built by extending the non-reducing end of the bound cellotetraose with another glucosyl monomer (41). For Athe_0598, glucose was positioned by homology to 5SWI; cellobiose was positioned in the sites aligned with 2O71; cellotriose was initially positioned by homology to 4G68; cellotriose was positioned in subsites from 7C68; and cellopentaose was positioned using the aligned subsites in 7C6R (41, 58–60). Two hundred conformers of each carbohydrate substrate were generated using BCL (67). Each protein–substrate combination yielded 200 docked structures, and interface energies of binding were quantified from the 10 lowest energy structures. KDEEP, a 3D convolutional neural network method for assessing interface energy, was used to further evaluate the ligand-bound pose (68). All protein structures were visualized using PyMOL (69).

## SUPPLEMENTAL MATERIAL

Supplemental material aem.01284-25-s0001.pdf Figures S1 to S3; Tables S1 to S3.

ASM does not own the copyrights to Supplemental Material that may be linked to, or accessed through, an article. The authors have granted ASM a non-exclusive, world-wide license to publish the Supplemental Material files. Please contact the corresponding author directly for reuse.
