# Supplementary material for: A highly conserved ABC transporter mediates cello-oligosaccharide uptake in the extremely thermophilic, lignocellulolytic bacterium Anaerocellum bescii (f. Caldicellulosiruptor bescii)
Source: Appl Environ Microbiol. 2025 Dec 18;92(1):e01284-25. doi: 10.1128/aem.01284-25 (PMC12838199; doi:10.1128/aem.01284-25)
Supplement: Supplemental material — Figures S1 to S3; Tables S1 to S3. [file aem.01284-25-s0001.pdf]

Supplemental material for:

A highly conserved ABC transporter mediates cello-oligosaccharide uptake in the extremely thermophilic, lignocellulolytic bacterium *Anaerocellum bescii* (f. *Caldicellulosiruptor bescii*)

Hansen Tjo [1], Virginia Jiang [1], Anherutowa Calvo [1], Jerelle A. Joseph [1,2], Jonathan M. Conway [1,2,3,4,5,#]

Author Affiliations:

[1] Department of Chemical and Biological Engineering, Princeton University, Princeton, NJ 08544, USA

[2] Omenn-Darling Bioengineering Institute, Princeton University, Princeton, NJ 08544, USA

[3] Molecular Biology Department, Princeton University, Princeton, NJ 08544, USA

[4] Andlinger Center for Energy and the Environment, Princeton University, Princeton, NJ 08544, USA

[5] High Meadows Environmental Institute, Princeton University, Princeton, NJ 08544, USA

#Corresponding Author: Jonathan M. Conway, jmconway@princeton.edu

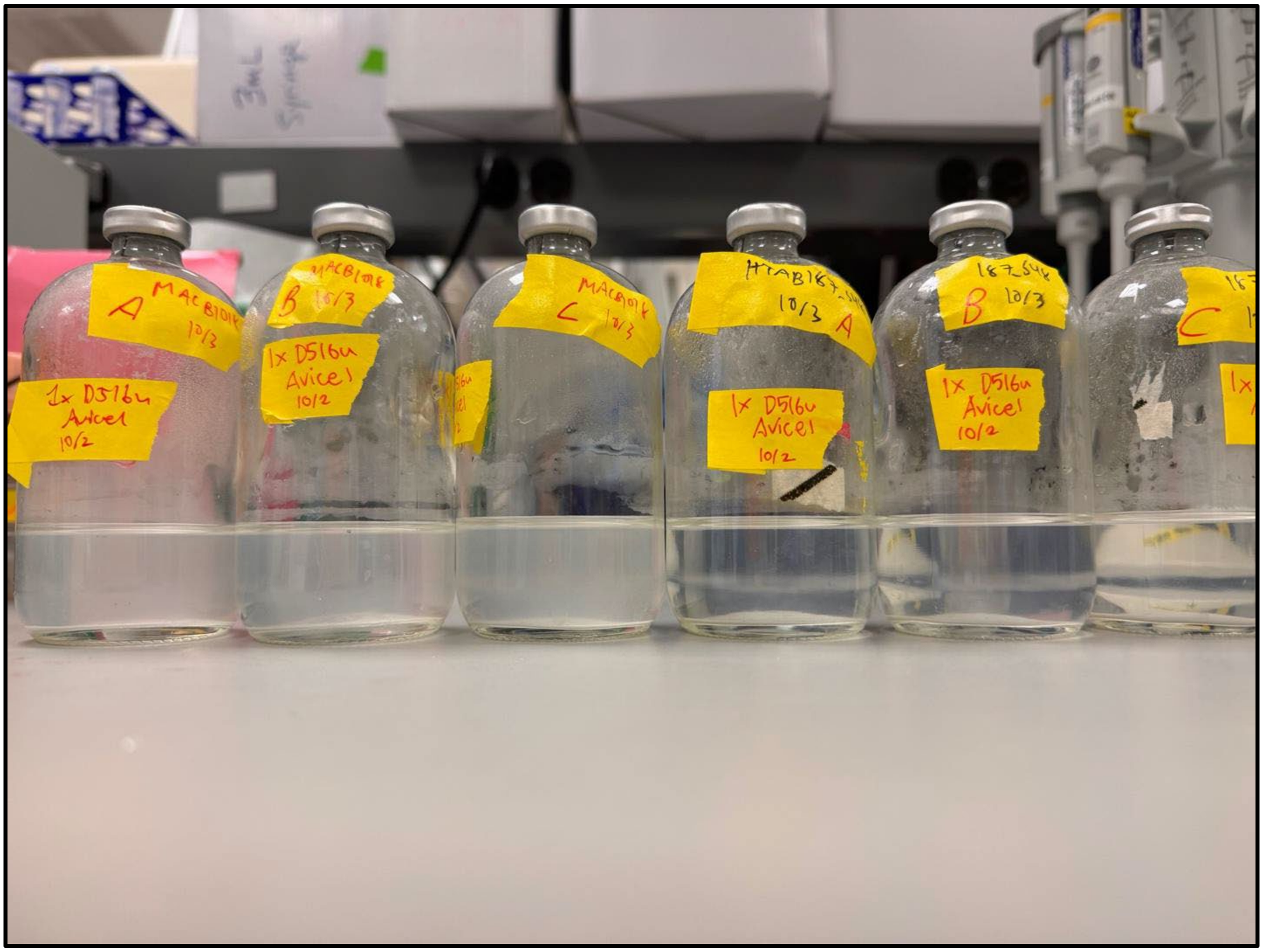

Figure S1: Visual illustration of parent strain MACB1018 ($\Delta pyrE$) (the three bottles on the left) and engineered strain HTAB187 ($\Delta pyrE\ \Delta athe\_0595 - 0598$) (the three bottles on the right) grown on DSM 516 medium with microcrystalline cellulose growth substrate after 40 hours, in biological triplicate. Visually, bottles inoculated with MACB1018 contain more turbid cultures compared to bottles inoculated with HTAB187.

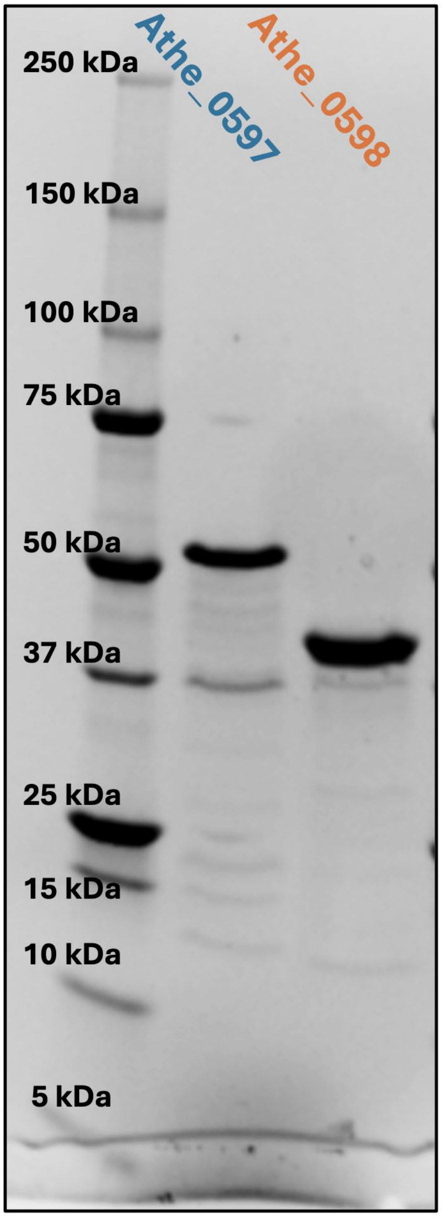

Figure S2: Purified Athe_0597 and Athe_0598, without their respective signal peptides, run on SDS-PAGE.

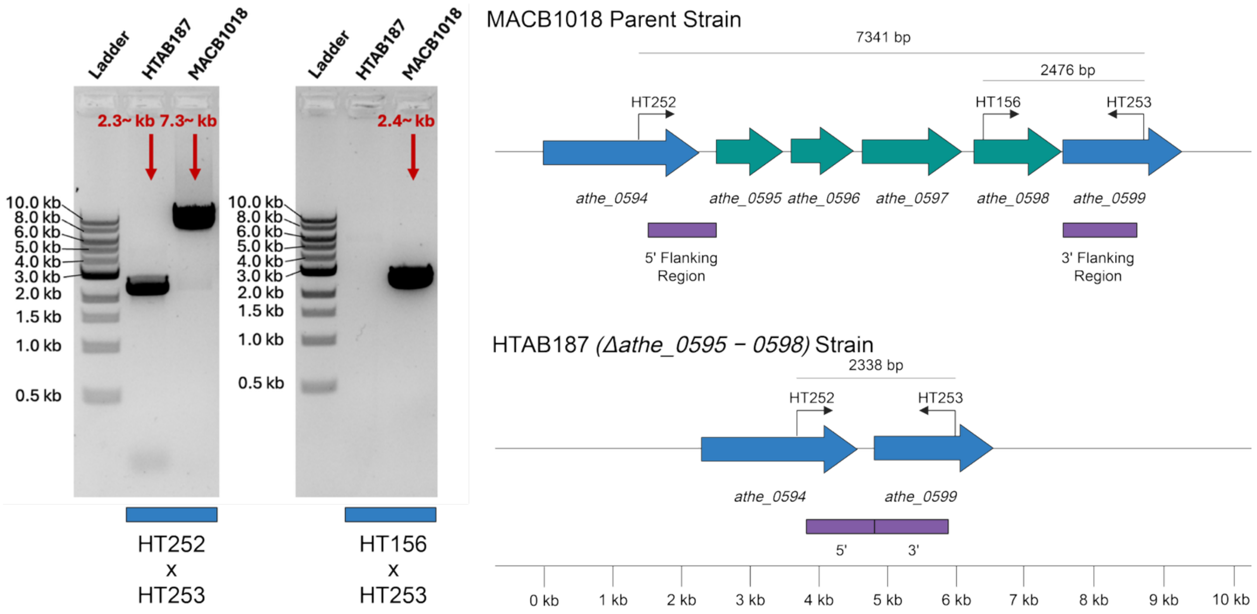

Figure S3: PCR verification that the gene locus *athe_0595* – *athe*_0598 was deleted in the genomic DNA of engineered *A. bescii* strain HTAB187, in comparison to genomic DNA from parent strain MACB1018 as negative control.

Table S1: Table of results from docking cello-oligosaccharide substrates and glucose onto Athe_0597 and Athe_0598. Each protein-ligand combination was simulated two hundred times ($n = 200$).

	Athe_0597		Athe_0598	
Ligand	ΔG (kcal/mol)	Standard Deviation (kcal/mol)	ΔG (kcal/mol)	Standard Deviation (kcal/mol)
Glucose (G1)	-4.7944	0.7765	-5.6253	0.4796
Cellobiose (G2)	-7.0972	0.7984	-14.0418	0.5927
Cellotriose (G3)	-7.5338	0.9405	-7.8047	1.9189
Cellotetraose (G4)	-9.8648	1.1934	-2.6703	2.8200
Cellopentaose (G5)	-8.7800	1.0898	-2.4503	0.7175

Table S2: Table of Primers used in this study.

Primer	Sequence (5' – 3')	Application
HT001_F	GCTGCCACCGCTGAGCAATAACTAG	pRGB001 Vector.FOR
HT002_F	CTTGTCGTCGTCATCCACGTGATG	pRGB001 Vector.REV
HT015	TCACGTGGATGACGACGACAAGTCAAAACTTCCTTATGTTAAGCTTACATGGTATGTC	Athe_0597 FOR Primer
HT016	GTTATTGCTCAGCGGTGGCAGCTTATTTCTTTCTTCCTGTCTTCTTCAGAAATTCATC	Athe_0597 REV Primer
HT156	TTACTTCCAGGGCGCCATGAGTGTAACTATTGAAAATGAAGAGTTAGAAG	Athe_0598 FOR Primer
HT157	TTGTTAGCAGCCGGATCTCATCATTCACCTAATTTCTCCATATTCTC	Athe_0598 REV Primer
HT232	GCGGCGCGCCGTTTAAACGCTGGGCTCAGGTACAAGACAG	5' Flank FOR Primer
HT233	TTTTAACATTTTCATTGATGCCGTCTCCTTTCTCAAGTTG	5' Flank REV Primer
HT234	TGAGAAAGGAGACGGCATCAATGAAAATGTTAAAAATAAAACTTATTTTTAG	3' Flank FOR Primer
HT235	GCTGGCCTTTTGCTCACATACTTTTAGTTCTGCTTCTTTTCG	3' Flank REV Primer
HT110	GACTTGAGCGTCGATTTTTGTGATG	colE1 REV Primer to verify 1x crossovers in conjunction with HT252
HT252	ACCAGATAGGGCTTCAAAAGG	FOR Primer to verify Athe_0595 – 0598 Clean DEletion
HT253	AGCTGTGCAAGCCAGTTAATCG	REV Primer to verify Athe_0595 – 0598 Clean DEletion

Table S3: Sequences of all proteins analyzed in this study. Residues comprising the signal peptide sequence are marked in red. Only sequences obtained from AlphaFold 2.0 contain signal peptides.

Primer	Amino Acid Sequence
Athe_0597<br>(AlphaFold 2.0 Sequence)	MNLKKFFVVMLVVTFVLTSVIGVVTGFGASSSKLPYVKLTWYVIGTPQKDWDLINQKV<br>NEYIKPKLNAEIKMTMFDWGEYNDKLQTKIAASEPFDICFTAIWTNNYRTNVAKGAFLP<br>LNKPGNDLLSKYAPKTKKLLGDDFIKGASINGILYAIPANKEKAHNWGFIVRMDLVKKYK<br>LEDMFKKVKKLEDLEPYLKVIKQKEPGVYPLGAYAGESPRFLLDWDKVVDDDVPVSLYP<br>NNKSTKIVNELEQPNTKALFKTVRKYYLAGYIRKDAASVTDWMSDLKAGKVFVMPQSL<br>KPGKDAEMSISTGYEWKQIDITPPVMSTRECIGSMQAINAKSKNPERALMFLELFNTDK<br>YLNNLVNFGIEGQHYVFKDKARGIIAPGPKAKDYSPGLGWMFGNQFINYIYENEDPNK<br>WKNFEEYNKKALPLLSLGFNFDDSKVKTQVAACKSVWKQYIPMLETGSVDPDKYIPQAI<br>DKFKKAGVDIIIKEAQKQYDEFLKKTGRKK
Athe_0598<br>(AlphaFold 2.0 Sequence)	MNDSFNKRRLLSISFLVPLVTTLVLIIVLILNTQKTIEESVTIENEELEVNTKIRFLSPWGGSD<br>PYAETLSFVLQKFQEENPGVTIVNESLFGDDFLIKLQTDFASGNPPDVFGLFPGSVRDLLI<br>KRKQIAELTNILKKDVKWYQSFYSNMWKYVTFNGKIYGVPLETIVECLFVNKDIFEKYNL<br>KVPQTLDDLISVSKILKSKGIIPIAFNAQPEGTYIYQNIIVSIGTKYEVENPIKNGEFSLPYIKA<br>LDYLKVLYKAGAFPANYYSLTSKQRNDLFLTKKAAMIVQGSWFIPKCDPKTVDIYIFPQA<br>NEKGKKHLIYGLGAGTFYVSSQAWQDIEKRNSAIKLLKFLSSEKIARIFVERTGLISNVKIK<br>NPPNVKNSLRSKVEGLIKEADVLVAPPDHFVDRMVWEEVITKNIPYYLQGTISSKLFWA<br>RAVKAWKENMEKLGE
Athe_0597 (purified)	MAHHHHHHVDDDDKSKLPYVKLTWYVIGTPQKDWDLINQKVNEYIKPKLNAEIKMT<br>MFDWGEYNDKLQTKIAASEPFDICFTAIWTNNYRTNVAKGAFLPLNKPGNDLLSKYAPK<br>TKKLLGDDFIKGASINGILYAIPANKEKAHNWGFIVRMDLVKKYKLEDMFKKVKKLEDLE<br>PYLKVIKQKEPGVYPLGAYAGESPRFLLDWDKVVDDDVPVSLYPNNKSTKIVNELEQPN<br>TKALFKTVRKYYLAGYIRKDAASVTDWMSDLKAGKVFVMPQSLKPGKDAEMSISTGYE<br>WKQIDITPPVMSTRECIGSMQAINAKSKNPERALMFLELFNTDKYLNNLVNFGIEGQHY<br>VFKDKARGIIAPGPKAKDYSPGLGWMFGNQFINYIYENEDPNKWKNFEEYNKKALPLLS<br>LGFNFDDSKVKTQVAACKSVWKQYIPMLETGSVDPDKYIPQAIDKFKKAGVDIIIKEAQK<br>QYDEFLKKTGRKK
Athe_0598 (purified)	MGSSHHHHHHSSGENLYFQGAMSVTIENEELEVNTKIRFLSPWGGSDPYAETLSFVLQ<br>KFQEENPGVTIVNESLFGDDFLIKLQTDFASGNPPDVFGLFPGSVRDLLIKRKQIAELTNIL<br>KKDVKWYQSFYSNMWKYVTFNGKIYGVPLETIVECLFVNKDIFEKYNLKVPQTLDDLISV<br>SKILKSKGIIPIAFNAQPEGTYIYQNIIVSIGTKYEVENPIKNGEFSLPYIKALDYLKVLYKAGA<br>FPANYYSLTSKQRNDLFLTKKAAMIVQGSWFIPKCDPKTVDIYIFPQANEKGKKHLIYGL<br>GAGTFYVSSQAWQDIEKRNSAIKLLKFLSSEKIARIFVERTGLISNVKIKNPPNVKNSLRSK<br>VEGLIKEADVLVAPPDHFVDRMVWEEVITKNIPYYLQGTISSKLFWARAVKAWKENME<br>KLGE
PDB: 5SUO	KPVIKMYQIGDKPDNLDELLANANKIIEEKVGAKLDIQYLGWGDYGKKMSVITSSGENY<br>DIAFADNYIVNAQKGAYADLTELYKKEGKDLYKALDPAYIKGNTVNGKIYAVPVAANVA<br>SSQNFAFNGTLLAKYGIDISGVTSYETLEPVLKQIKEKAPDVVPFAIGKVFIPSDNFDYPVA<br>NGLPFVIDLEGDTTKVVNRYEVPRFKEHLKTLHKFYEAGYIPKDVATSDTSFDLQQDTWF<br>VREETVGPADYGNSLLSRVANKDIQIKPITNFIKKNQTTQVANFVISNNSKNKEKSMEIL<br>NLLNTNPELLNGLVYGPEGKNWEKIEGKENRVRVLDGYKGNTHMGGWNTGNNWILY<br>INENVTDQQIENSKKELAEAKESPALGFIFNTDNVKSEISAIANTMQQFDTAINTGTVDP<br>DKAIPELMEKLKSEGAYEKVLNEMQKQYDEFLKNKK
PDB: 5SWA	MGNLTGNSKKAADSGDKPVIKMYQIGDKPDNLDELLANANKIIEEKVGAKLDIQYLGW<br>GDYGKKMSVITSSGENYDIAFADNYIVNAQKGAYADLTELYKKEGKDLYKALDPAYIKGN<br>TVNGKIYAVPVAANVASSQNFAFNGTLLAKYGIDISGVTSYETLEPVLKQIKEKAPDVVPF<br>AIGKVFIPSDNFDYPVANGLPFVIDLEGDTTKVVNRYEVPRFKEHLKTLHKFYEAGYIPKD<br>VATSDTSFDLQQDTWFVREETVGPADYGNSLLSRVANKDIQIKPITNFIKKNQTTQVAN

	FVISNNSKNKEKSMEILNLLNTNPELLNGLVYGPEGKNWEKIEGKENRVRVLDGYKGNT<br>HMGGWNTGNNWILYINENVTDQQIENSKKELAEAKESPALGFIFNTDNVKSEISAIANT<br>MQQFDTAINTGTVDPDKAIPELMEKLKSEGAYEKVLNEMQKQYDEFLKNKKLEHHHH<br>HH
PDB: 5SWB	MGNLTGNSKKAADSGDKPVIKMYQIGDKPDNLDELLANANKIIEEKVGAKLDIQYLGW<br>GDYGKKMSVITSSGENYDIAFADNYIVNAQKGAYADLTELYKKEGKDLYKALDPAYIKGN<br>TVNGKIYAVPVAANVASSQNFAFNGTLLAKYGIDISGVTSYETLEPVLKQIKEKAPDVVPF<br>AIGKVFIPSDNFDYPVANGLPFVIDLEGDTTKVVNRYEVPRFKEHLKTLHKFYEAGYIPKD<br>VATSDTSFDLQQDTWFVREETVGPADYGNSLLSRVANKDIQIKPITNFIKKNQTTQVAN<br>FVISNNSKNKEKSMEILNLLNTNPELLNGLVYGPEGKNWEKIEGKENRVRVLDGYKGNT<br>HMGGWNTGNNWILYINENVTDQQIENSKKELAEAKESPALGFIFNTDNVKSEISAIANT<br>MQQFDTAINTGTVDPDKAIPELMEKLKSEGAYEKVLNEMQKQYDEFLKNKKLEHHHH<br>HH
PDB: 4G68	MAHHHHHHVDDDDKMCSSNNLSKSNTSNSSKTSSSSKKMCSSNNLSKSNTSNSSKTSS<br>SSKKITLTFWNLFTGEPAKTKVKEIIDQWNKENPNVQIVESVTENDAYKTKIKAAIAANE<br>APDIFQTWAGGFSQPFVEAGKVLQLDSYLNDGTKDQLLPGSFDNVTYNGKIYGIPFDQ<br>QASVLYINKELFDKYNVKVPTTFSELIDAIKTFKSKGVTPFALGEKDEWPGMWYYDMIAL<br>REGGVQLTRDALNGKASFDNQAFTDAAQKLQDMVNAGAFDSGFMGLTRDEATAEFN<br>QGKAAMYFGGNFDAAAFVSDPSSLVKGKIEAVRFPTIEGGKGDPTEYIGGTVGALMVS<br>ANSKYKDEAVRAAKYLAKQLSDMDYLIATGLPAWKYDNIDQSKVDPLEIQIMNNIVAN<br>AKGSVPAWDIYLSGDAAQTHKDLVAQLFAKQITPEEYSKQMQQKINGK
PDB: 7EHP	MTVSLRHTQVRDDVRLRLKMLEDIAQRMEAAVPGLRVELEGVEDKVNRFEKLPAEMA<br>AGNPPKIFDLFGGTDTAKYVKAGRLLELTPILNELGLKDKFPNLQEFTVDGKIYGLPTAYF<br>VEGVFYNKQIFKQLNVDVPRRWEDLMDVAAKAKASGFVPFAFASSDGWVANMMLN<br>TLWVRTAGDDSVPGFVRGTRRWTDPDVADGFKRYDTLLKKGYLQEGSLGQKYAEQQY<br>AFREGRAAMMFDGSWASAALVDAGKTKIAEDIGFFSFPDVGGKGDGMINGGYSNGY<br>GFSASLNEREKKAAVEFIKIMYSEEMQKRQLKESGILPAMKLSDLSGVHPVIREMIQASE<br>LRQFPAFDSIVQAKVRETLEMCMQELIGGRMTVEQVLDKMQKVQEDANRDMKKLEH<br>HHHHH
PDB: 2O7I	MQVSLPREDTVYIGGALWGPATTWNLYAPQSTWGTDQFMYLPAFQYDLGRDAWIPV<br>IAERYEFVDDKTLRIYIRPEARWSDGVPITADDFVYALELTKELGIGPGGGWDTYIEYVKA<br>VDTKVVEFKAKEENLNYFQFLSYSLGAQPMPKHVYERIRAQMNIKDWINDKPEEQVVS<br>GPYKLYYYDPNIVVYQRVDDWWGKDIFGLPRPKYLAHVIYKDNPSASLAFERGDIDWN<br>GLFIPSVWELWEKKGLPVGTWYKKEPYFIPDGVGFVYVNNTKPGLSDPAVRKAIAYAIPY<br>NEMLKKAYFGYGSQAHPSMVIDLFEPYKQYIDYELAKKTFGTEDGRIPFDLDMANKILD<br>EAGYKKGPDGVRVGPDGTKLGPYTISVPYGWTDWMMMCEMIAKNLRSIGIDVKTEFP<br>DFSVWADRMTKGTFDLIISWSVGPSFDHPFNIYRFVLDKRLSKPVGEVTWAGDWERYD<br>NDEVVELLDKAVSTLDPEVRKQAYFRIQQIIYRDMPSIPAFYTAHWYEYSTKYWINWPS<br>EDNPAWFRPSPWHADAWPTLFIISKKSDPQPVPSWLGTVDEGGIEIPTAKIFEDLQKAT<br>MHHHHHH
